# Prescription of antiviral drugs during the 2009 influenza pandemic: an observational study using electronic medical files of general practitioners in the Netherlands

**DOI:** 10.1186/2050-6511-14-55

**Published:** 2013-10-21

**Authors:** Mariëtte Hooiveld, Tine van de Groep, Theo JM Verheij, Marianne AB van der Sande, Robert A Verheij, Margot AJB Tacken, Gerrit A van Essen

**Affiliations:** 1 NIVEL, Netherlands Institute for Health Services Research, Utrecht, PO Box 1568, 3500 BN Utrecht, the Netherlands; 2 Julius Center for Health Sciences and Primary Care, University Medical Center Utrecht, Utrecht, the Netherlands; 3 Centre for Infectious Disease Control, National Institute for Public Health and the Environment, Bilthoven, the Netherlands; 4 IQ healthcare, Scientific Institute for Quality of Healthcare, Radboud University Nijmegen Medical Centre, Nijmegen, the Netherlands

**Keywords:** Drug prescriptions, General practice, Influenza, Human, Antivirals, Pandemics, Practice guidelines as topic

## Abstract

**Background:**

After the clinical impact of the A(H1N1) pdm09 virus was considered to be mild, treatment with antiviral drugs was recommended only to patients who were at risk for severe disease or who had a complicated course of influenza. We investigated to what extent antiviral prescriptions in primary care practices were in accordance with the recommendations, what proportion of patients diagnosed with influenza had been prescribed antiviral drugs, and to what extent prescriptions related to the stated indications for antiviral treatment.

**Methods:**

We used data from routine electronic medical records of practices participating in the Netherlands Information Network of General Practice LINH in the period August - December 2009. We considered patient and practice characteristics, clinical diagnoses and drug prescriptions of all patients who contacted their general practitioner in the given period and who had been prescribed antiviral medication (n = 351) or were diagnosed with influenza (n = 3293).

**Results:**

Of all antiviral prescriptions, 69% were in accordance with the recommendations. Only 5% of patients diagnosed with influenza were prescribed antiviral drugs. This percentage increased to 12% among influenza patients belonging to the designated high risk groups. On the other hand, 2.5% of influenza patients not at high risk of complications received antiviral treatment. In addition to the established high risk factors, the total number of drug prescriptions for a patient in this year was a determinant of antiviral prescriptions. Information on time since onset of symptoms and the clinical presentation of patients was not available.

**Conclusions:**

General practitioners in the Netherlands have been restrictive in prescribing antiviral drugs during the influenza pandemic, even when patients met the criteria for antiviral treatment.

## Background

On April 30th 2009, the first case of influenza A(H1N1)pdm09 was identified in the Netherlands, while it eventually caused an epidemic with influenza activity above baseline in the period 12 October through 13 December 2009 (fall season in the northern hemisphere) [1].

During the emerging pandemic, all patients suspected of influenza were indicated for treatment with antiviral drugs and their relatives (household contacts) were eligible for prophylactic antiviral drug use. Antiviral drugs were sufficiently available from the national stockpile that has been established since the H5N1 outbreak in 2003. In the beginning of August 2009, the Health Council of the Netherlands and the National Institute for Public Health and the Environment (RIVM) concluded, in concordance with WHO recommendations [2], that the impact of the H1N1 virus on morbidity and mortality appeared to be similar to seasonal influenza virus, based on its so far documented relatively mild course [3,4]. Treatment with antiviral drugs was now only recommended for patients at risk for severe disease and patients having a complicated course of influenza [5]. Prophylactic prescriptions were no longer recommended. High risk patients were defined as the same patients eligible for seasonal influenza vaccination, *i.e.* all patients of 60 years or above and patients with certain medical conditions [5,6]. In addition to that, children under two years of age, pregnant women in their third trimester, and patients having symptoms of influenza in whom the infection occurred to be unusually severe or with complications were considered [5]. In the Netherlands, oseltamivir was the drug of first choice for treatment of high risk patients suspected of influenza during the pandemic. Treatment with zanamivir was reserved for patients suspected for an infection with a resistant influenza virus. Oseltamivir was only available as a prescription drug, not over the counter, and it was in stock for approximately one third of the Dutch population (5 million doses for 16 million inhabitants).

In view of future epidemics, it is important to evaluate the implementation of and compliance to the recommended guidelines. The general aim of this study was therefore to investigate whether the prescription of oseltamivir by general practitioners (GPs) in the Netherlands was in accordance with the national guidelines described above. We used data from a large national database to estimate 1) the proportion of patients receiving oseltamivir according to the guidelines in relation to patients’ and practices’ characteristics, and 2) what proportion of patients diagnosed with influenza received oseltamivir and which factors determined this prescription.

## Methods

### Practices and patients selection

Data for this study were derived from the routine electronic medical records of GP practices that participate in the Netherlands Information Network of General Practice (LINH), a national network of around 90 general practices, who are representative of all Dutch general practices with respect to geographical distribution and degree of urbanization [7]. All Dutch citizens are enlisted as patients in a family practice, so the population listed in a general practice can be used as the denominator in epidemiological studies. The GP is the first professional to contact for health problems and a referral to the secondary health care system (hospitals and medical specialists) can only be made by the GP. The LINH practice population consists of more than 350,000 registered patients, representative of the Dutch population with respect to age and gender. The database holds longitudinal data on morbidity, prescriptions and referrals. Clinical diagnoses are coded using the ICPC (International Classification of Primary Care) coding system [8]. Drugs are coded according to the Anatomical Therapeutic Chemical (ATC) classification [9]. GP practices were included when they provided data on the registration of claimed services, ICPC-coded diagnoses, and ATC-coded prescriptions for the full calendar year of 2009. Only patients enlisted with the general practice during the whole year 2009 were included. Data collection within the LINH network is carried our according to Dutch legislation on privacy. Each patient is coded with an anonymous administrative number. The key to this coding number is only with the general practitioner. The privacy regulation of the LINH network was approved by the Dutch Data Protection Authority. According to the Dutch Central Committee on Research Involving Human Subjects, obtaining informed consent is not obligatory for observational studies and approval by the Medical Ethical Committee was not necessary.

### Data collection

All contacts (claims) of patients with the GPs were considered. First, patients having a prescription of oseltamivir (ATC-code J05AH02) between August 8 and December 31, 2009 were considered. No prescriptions of zanamivir were registered. When there was more than one oseltamivir prescription, only the first one was taken into account. We looked for all diagnoses within 7 days before or after the date of prescription, in order to take into account registration delays. However, in 88% of oseltamivir prescriptions, the diagnosis was registered at the same day. We specifically considered diagnoses of influenza (ICPC-code R80, which will be used by GPs for patients with influenza-like illness in the context of an influenza epidemic, or upon virological confirmation of an influenza virus), another acute respiratory infection (ARI, defined by ICPC-codes R74 - acute upper respiratory infection, R78 - acute bronchitis/bronchiolitis or R81 - pneumonia), and non-specified virus infection (A77, which can be used by GPs for patients with influenza-like illness outside the influenza season).

Patients who did not receive oseltamivir were evaluated for a diagnosis of influenza. When more than one episode was present, the date of the first consultation was taken. Recommended treatment of high risk patients with oseltamivir was based on age (<2 years or ≥60 years), co-morbidity (cardiac disease, respiratory disease, diabetes mellitus, chronic renal insufficiency, reduced resistance against infections and children up to 18 years using salicylates), a complicated course of illness (the occurrence of pneumonia within 7 days before or after the diagnosis of influenza or ARI, or the need to prescribe antibiotics – not for urinary infections – within these days), or a third trimester pregnancy (an oseltamivir prescription or an influenza diagnosis between 20 to 34 weeks after a first contact related to pregnancy, based on the assumption that pregnant women usually have their first pregnancy-related contact with their GP between weeks 6 and 20; a contact indicating the birth of a child had to be no more than 14 weeks after oseltamivir prescription or influenza diagnosis). Details on ICPC- and ATC-codes used to classify high-risk patients have been published elsewhere [10].

Additional patient characteristics comprised gender, month of prescribing, total number of contacts with the practice in 2009, and the total number of prescriptions in 2009 besides oseltamivir. Other underlying chronic health conditions were based on a selection of diseases with a high prevalence, a long-term course and a serious illness, as used in the National Public Health Compass (http://www.nationaalkompas.nl). This selection is based on the list of chronic conditions from the Australian Family Medicine Research Centre (http://www.fmrc.org.au) and adapted from ICPC-2 to ICPC-1. Practice characteristics included practice type, dispensing practice, and level of urbanisation and geographic region of the practice location.

### Statistical analysis

In the group of patients with a diagnosis of influenza, we evaluated the proportion of patients whom had been prescribed oseltamivir and whom should have been prescribed it. Univariate logistic regression analyses were performed on the association between the prescription of oseltamivir and a recommendation for a prescription, as well as other patients’ and practices’ characteristics. Multivariable regression analyses were performed to assess potential determinants independently associated with the prescription of oseltamivir. Likewise, we evaluated whether oseltamivir was prescribed according to the guidelines among all patients receiving oseltamivir. Because of the recommendation to prescribe oseltamivir to both very young (under two years of age) and old (60 years or above) patients, we added age-squared in the multivariable models to take into account the u-shaped association when using the continuous age variable. Only variables that significantly improved the model fit, based on Likelihood-ratio tests (*p*-value < 0.05), were included. Since patients were clustered within practices, we used multilevel logistic regression. All analyses were performed in Stata version 11.2 (StataCorp LP, College Station, TX, USA).

## Results

We included 68 GP practices with complete and reliable data records for the year 2009. Twelve patients with missing age were excluded, leaving 260,298 enlisted patients for analyses (1.6% of the Dutch population). Of all patients, 351 (1.4 per 1,000 enlisted patients) had been prescribed oseltamivir. The total number of oseltamivir prescriptions ranged between practices from 0 (11 practices) to 10.7 prescriptions per 1000 enlisted patients (right-skewed distribution; geometric mean = 0.9; geometric standard distribution = 3.4).

Half of the patients receiving oseltamivir had a diagnosis of influenza (n = 181; 51.6%). The most frequently registered diagnoses in 105 patients not having influenza, ARI or a non-specified viral infection were general disease (21 patients), fever (17 patients), other viral disease (12 patients), and ‘no disease’ (11 patients). No valid ICPC-code was recorded for 23 patients (6.6%). Of all oseltamivir prescriptions, 241 (68.7%) were to patients belonging to the designated high risk groups. Table 1 shows the distribution of the underlying reasons for qualification for oseltamivir in these patients.

**Table 1:** Characteristics of 241 patients at high risk of severe illness who had been prescribed oseltamivir

	***n *of patients**	**(%)**
**High risk group description**		
Age only	53	(22.0)
Co-morbidity only	101	(41.9)
Complicated course of illness only	13	(5.4)
Age and co-morbidity	49	(20.3)
Age and complicated course of illness	2	(0.8)
Co-morbidity and complicated course	15	(6.2)
Age, co-morbidity and complicated course	8	(3.3)
Specific co-morbidity at risk for complications*		
Cardiac disease	60	(24.9)
Respiratory disease	106	(44.0)
Diabetes mellitus	27	(11.2)
Chronic renal insufficiency	5	(2.1)
Reduced resistance against infections	24	(10.0)
Children using salicylates	1	(0.4)
Third trimester of pregnancy	3	(1.2)

*Numbers add up above 100% because of multiple co-morbidities per patient.

Table 2 shows factors that independently influenced a prescription according to clinical guidelines among all patients who had been prescribed oseltamivir. As expected, both a very young and a higher age were associated with an increased chance of having an oseltamivir prescription according to the recommendations. Independently of age, a higher number of contacts with the GP and a higher number of prescriptions in general were positively associated with prescribing oseltamivir according to the guidelines.

**Table 2:** Factors influencing a prescription of oseltamivir according to clinical guidelines in 351 patients with antiviral medication

	**Univariate analysis**		**Adjusted multilevel analysis**		
Variable	OR	(95% CI)	OR	(95% CI)	*P*-value
Age at prescription (continuous)	0.87	(0.83 to 0.91)	0.84	(0.80 to 0.90)	<0.001
Age-squared	1.00	(1.00 to 1.00)	1.00	(1.00 to 1.00)	<0.001
Number of contacts with GP in 2009					
<6	ref	-	ref	-	
6 or more	3.00	(1.87 to 4.82)	2.19	(1.18 to 4.08)	0.014
Total number of prescriptions in 2009*					
<8	ref	-	ref	-	
8 or more	4.60	(2.80 to 7.57)	3.24	(1.63 to 6.42)	0.001

GP = general practitioner; OR = odds ratio; CI = confidence interval.

*Excluding oseltamivir prescriptions.

A total of 3,293 patients (1.3% of the study population) were diagnosed with influenza and oseltamivir was prescribed to 181 (5.5%). The weekly number of influenza patients followed well the epidemiologic curve of the Dutch influenza-like illness (IAZ) surveillance (data not shown) [1]. Of all influenza patients, 1,051 (31.9%) belonged to the designated high risk groups, of whom 126 (12.0%) indeed received oseltamivir. This percentage varied from 9.5% (*n* = 16) among children aged 2 to 14 years to 18.0% (*n* = 18) among children under two years of age. On the other hand, 2,242 patients diagnosed with influenza did not belong to the high risk groups and yet 55 (2.5%) received a prescription of oseltamivir.

Univariate analyses showed that age, underlying co-morbidity, the total number of contacts with the GP in 2009 and the total number of prescriptions in 2009 were determinants of the prescription of oseltamivir among influenza patients (Table 3). Patients with underlying co-morbidity had the highest chance of an oseltamivir prescription. All of the designated high risk groups were statistically significantly associated with oseltamivir prescription, except children receiving chronic aspirin therapy. Taking into account specific medical conditions showed that patients with respiratory diseases and pregnant women had a higher chance of treatment, while no associations were observed for other underlying diseases. Ten percent of all influenza patients had a complicated course of illness, but this was not associated with oseltamivir treatment. Practice characteristics were not associated with oseltamivir prescriptions.

**Table 3:** Potential factors influencing the prescription of oseltamivir by general practitioners to 3,293 influenza patients

	**Prescription of oseltamivir, *n *of patients (%)**				**Univariate analysis**		**Adjusted multilevel analysis**	
Variable	No		Yes		OR	(95% CI)	OR	(95% CI)
Gender							*(not in model)*	
Male	1,477	(47.5)	88	(48.6)	*ref*	-		
Female	1,635	(52.5)	93	(51.4)	0.94	(0.70 to 1.28)		
Age at diagnosis								
< 2 yrs	82	(2.6)	18	(9.9)	3.62	(2.07 to 6.36)	5.71	(3.01 to 10.80)
2 to 14 yrs	972	(31.2)	32	(17.8)	0.54	(0.36 to 0.83)	0.70	(0.44 to 1.11)
15 to 24 yrs	469	(15.1)	20	(11.0)	0.70	(0.43 to 1.16)	0.88	(0.51 to 1.52)
25 to 59 yrs	1,321	(42.4)	80	(44.2)	*ref*	-	*ref*	-
60 yrs or older	268	(8.6)	31	(17.1)	1.84	(1.19 to 2.87)	0.96	(0.58 to 1.57)
Co-morbidity at risk for complications								
No	2,562	(82.3)	89	(49.2)	*ref*	-	*ref*	-
Yes	550	(17.7)	92	(50.8)	4.82	(3.53 to 6.57)	4.94	(3.29 to 7.40)
Number of co-morbidities at risk							*(not in model)*	
0	2,562	(82.3)	89	(49.2)	*ref*	-		
1	443	(14.2)	70	(38.7)	4.55	(3.27 to 6.32)		
2 or 3	107	(3.4)	22	(12.1)	5.92	(3.57 to 9.81)		
Specific co-morbidity at risk for complications*							*(not in model)*	
Cardiac disease	178	(5.7)	28	(15.6)	3.04	(1.97 to 4.68)		
Respiratory disease	290	(9.3)	54	(30.0)	4.17	(2.95 to 5.89)		
Diabetes mellitus	112	(3.6)	17	(9.4)	2.79	(1.64 to 4.77)		
Chronic renal insufficiency	9	(0.3)	3	(1.7)	5.84	(1.57 to 21.82)		
Reduced resistance against infections	59	(1.9)	9	(5.0)	2.72	(1.33 to 5.59)		
Children using salicylates	13	(0.4)	1	(0.6)	1.33	(0.17 to 10.24)		
Third trimester of pregnancy	12	(0.4)	3	(1.7)	4.38	(1.22 to 15.68)		
Complicated course of illness								
No	2,810	(90.3)	159	(87.8)	*ref*	-	*ref*	-
Yes	302	(9.7)	22	(12.2)	1.29	(0.81 to 2.04)	0.95	(0.57 to 1.58)
Contacts with GP in 2009						-	*(not in model)*	
<6	1,850	(59.4)	86	(47.5)	*ref*			
6 or more	1,262	(40.6)	95	(52.5)	1.62	(1.20 to 2.19)		
Prescriptions in 2009 †								
<8	2,300	(73.9)	92	(50.8)	*ref*	-	*ref*	-
8 or more	812	(26.1)	89	(49.2)	2.74	(2.03 to 3.71)	1.36	(0.89 to 2.09)

GP = general practitioner; OR = odds ratio; CI = confidence interval.

*Reference group does not have the specific co-morbidity.

†Excluding oseltamivir prescriptions.

The results of the multilevel model with age, co-morbidity, complicated course of illness and number of prescriptions, are presented in Table 3 as well. After adjustment for other factors, an age of 60 years or above in itself was not associated with oseltamivir prescription. Independently of a high risk for severe disease due to age, co-morbidity or a complicated course of illness, the total number of drug prescriptions was positively associated with the prescription of oseltamivir.

Among influenza patients who did not belong to the designated high risk groups, chronic health conditions other than those at high risk of severe illness were not associated with oseltamivir prescription, nor were age, gender, and consultation rate or practice characteristics (Table 4). The only significant determinant for oseltamivir prescriptions was the total number of drug prescriptions in 2009. The odds ratio for drug prescriptions did not change in the multilevel logistic model (OR = 2.28; 95% CI = 1.20 to 4.32).

**Table 4:** Potential factors influencing the prescription of oseltamivir to 2,242 influenza patients not at high risk

	**Prescription of oseltamivir, *n *of patients (%)**				**Univariate analysis**	
Variable	No		Yes		OR	(95% CI)
*Patient characteristics*						
Gender						
Male	1,039	(47.5)	29	(52.7)	*ref*	-
Female	1,148	(52.5)	26	(47.3)	0.81	(0.47 to 1.39)
Age at diagnosis						
2 to 14 yrs	819	(37.4)	16	(29.1)	0.69	(0.37 to 1.29)
15 to 24 yrs	411	(18.8)	12	(21.8)	1.03	(0.52 to 2.06)
25 to 59 yrs	957	(43.8)	27	(49.1)	*ref*	-
Chronic health condition not at high risk						
No	1,754	(80.2)	45	(81.8)	*ref*	-
Yes	433	(19.8)	10	(18.2)	0.90	(0.45 to 1.80)
Number of contacts with GP in 2009						
<6	1,468	(67.1)	36	(65.5)	*ref*	-
6 or more	719	(32.9)	19	(34.5)	1.08	(0.61 to 1.89)
Total number of prescriptions in 2009*						
<8	1,888	(86.3)	41	(74.5)	*ref*	-
8 or more	299	(13.7)	14	(25.5)	2.16	(1.16 to 4.00)
*Practice characteristics*						
Type of practice						
Solo practice	706	(32.3)	24	(43.6)	*ref*	-
Duo practice	437	(20.0)	8	(14.6)	0.54	(0.24 to 1.21)
Group practice	1,044	(47.7)	23	(41.8)	0.65	(0.36 to 1.16)
Dispensing practice	101	(4.6)	2	(3.6)	0.78	(0.19 to 3.24)
Urbanicity practice location						
(Very) strongly urban	1,116	(51.0)	27	(49.1)	0.84	(0.42 to 1.68)
Moderately urban	418	(19.1)	12	(21.8)	*ref*	-
Mildly/not urban	653	(29.9)	16	(29.1)	0.85	(0.40 to 1.82)

GP = general practitioner; OR = odds ratio; CI = confidence interval.

*Excluding oseltamivir prescriptions.

## Discussion

This study showed that general practitioners in the Netherlands have been restrained in prescribing oseltamivir during the influenza pandemic. Only 5% of all patients diagnosed with influenza were prescribed oseltamivir, and only 12% of those who were at high risk of severe illness. On the other hand, when GPs have prescribed oseltamivir, they have rather well followed the recommendations in the national guidelines. Only 2.5% of influenza patients not at high risk were prescribed oseltamivir, and of all antiviral drugs prescribed, 69% were for patients at high risk for severe disease. The total number of drug prescriptions in 2009 and the total number of GP consultations, which can be considered proxies for underlying morbidity, were determinants for oseltamivir prescription in addition to the high risk factors of co-morbidity, a very young or an older age.

### Strengths and limitations of this study

This study was conducted using a large database of patients, representative for the general Dutch population, providing a complete picture of morbidity and prescriptions. A limitation of this study was the lacking information on specific details on presented symptoms. More severely ill patients could have been hospitalized, but information on care provided outside the general practice was lacking in our data. This could have resulted in an underestimation in the number of patients with a more severe illness, causing a negative association with the prescription of oseltamivir.

Co-morbidity at high risk of complications was defined by ICPC- and ATC-codes that are used in general practices to select patients eligible for influenza vaccination [10,11]. This method has been used to monitor the Dutch influenza vaccination rate in different high risk groups for several years, using the LINH database, with stable and representative results [12,13]. We assumed these patients had at least one contact (claim) with their GP, resulting in at least one record with the specified codes. We think it is very unlikely that a patient with a certain chronic condition did not contact his or her general practitioner for an entire year, nor was prescribed any medication for this chronic condition during that year. Misclassification of patients with co-morbidity is therefore unlikely.

### Comparison with other studies

Our observations are in line with those from Fietjé and colleagues [14], who found that 85% of Dutch patients with an oseltamivir prescription belonged to the high risk groups. Their study was based on telephone interviews with patients who filled a prescription for oseltamivir through community pharmacists. Results from other countries on prescriptions of oseltamivir during the A(H1N1)pdm09 pandemic are scarce. In a study by Hersh and colleagues, antivirals were prescribed in 58% of influenza visits to US ambulatory physicians [15]. The prescription rate for patients younger than 2 years was 47% and 68% for patients 65 years or older. Information on underlying medical conditions was not available. A study from the UK focusing on 90 pregnant women presenting with influenza-like illness in primary care, 61% were prescribed antiviral drugs [16]. Forty-three women were in their third trimester, of whom 26 (60%) received antivirals. In a Chicago hospital, 65% of high-risk patients with influenza-like illness received oseltamivir treatment [17]. These figures are much higher than in our study.

The results of our study show that if oseltamivir was prescribed, it was in accordance with the guidelines in most cases. A study by Grol et al. showed that compliance to guidelines was rather good in Dutch primary care; recommendations were followed in, on average, 61% of the decisions [18]. Based on our research it can therefore be said that when general practitioners in the Netherlands prescribed oseltamivir, they followed the recommendations on the prescribing fairly well. But they often did not prescribe, even when oseltamivir was recommended. The availability of antiviral drugs was no problem, since they were available from the national stockpile.

A possible explanation for withholding oseltamivir to patients at higher risk of complications might have been a late presentation of the patient. From the Dutch influenza-like illness surveillance we know that patients wait on average 3 days before contacting a doctor for respiratory complaints [19], while treatment with antiviral drugs should start preferably within 48 hours of the onset of illness. We had no information on time since onset of symptoms, but awareness and anxiety about the on-going pandemic may have urged patients to contact their GP earlier than usually.

## Conclusions

In conclusion, this study showed that general practitioners in the Netherlands have been very restrained in prescribing antiviral drugs during the influenza pandemic. Where GPs have used antiviral medication, they have rather well followed the recommendations in the national guidelines. To our knowledge, this is the first study in which recommendations for prescribing oseltamivir in primary care has been evaluated in detail. More information is needed on the reasons for underuse of antiviral drugs in patients belonging to the designated high risk groups. We believe it is important to evaluate the implementation of recommended guidelines. In this way, in the case of future epidemics, the recommendations may be adjusted and can be applied even more effectively.

## Abbreviations

ARI: Acute respiratory infection other than influenza; ATC: Anatomical Therapeutic Chemical classification; CI: Confidence interval; GP: General practitioner; ICPC: International Classification of Primary Care; LINH: Netherlands Information Network of General Practice; OR: Odds ratio.

## Competing interests

The authors declare that they have no competing interests.

## Authors’ contributions

MH, RV, MT and GvE contributed to conception and design. MH and TvdG acquired and analysed the data and drafted the manuscript. All authors read, contributed to and approved the final manuscript.

## Pre-publication history

The pre-publication history for this paper can be accessed here:

http://www.biomedcentral.com/2050-6511/14/55/prepub
